# Idiopathic scoliosis and associated factors among school children: a school-based screening in Ethiopia

**DOI:** 10.1186/s13690-021-00633-0

**Published:** 2021-06-18

**Authors:** Moges Gashaw, Balamurugan Janakiraman, Gashaw Jember Belay

**Affiliations:** 1 Department of Physiotherapy, School of Medicine, College of Medicine and Health Sciences, University of Gondar comprehensive specialized hospital, P.O. Box: 196, Gondar, Ethiopia; 2 Department of Physiotherapy, School of Medicine, College of Health Sciences and Ayder Comprehensive Specialized Hospital, Mekelle University, Tigrai, Ethiopia

**Keywords:** Ethiopia, Scoliosis, Scoligauge, iPhone app, school children, Spinal screening

## Abstract

**Background:**

Early detection of scoliosis is a preface to prevent progression. In resource-constrained countries like Ethiopia, there is a need for a cost-effective reliable solution for screening. The surprising fact is that there is neither idiopathic scoliosis screening nor reporting of data from Ethiopia. This study aimed to identify the magnitude, associated factors of scoliosis among school children by using non-invasive and less expensive scoliometer Smartphone app and Adams forward bending test.

**Methods:**

A school-based cross-sectional survey was carried out from March to June 2019 at seven primary schools. Children were screened by using physical examination (Adams forward bend test) and scoligauge iPhone app. Univariate and multivariable binary logistic regression model analysis was used to identify factors associated with idiopathic scoliosis.

**Results:**

A total of 1905 children aged between 5 and 16 years were screened, 3.3 % (95 % CI 2.5–4.1) of them were found positive using the scoligauge HD and among them only 1.8 % were identified positive for Adam’s forward bend test. The associated factors of idiopathic scoliosis identified by multivariate analysis were; spinal pain (AOR 4.1, 95 % CI 2.42, 6.89), student sitting on stool: (AOR, 7.03; 95 % CI, 1.52, 32.5), sitting on the bench with a backrest (AOR 4.12; 95 %CI, 1.12, 15.14) and sitting on the bench without backrest: (AOR 4.56; 95 % CI 1.20, 17.34).

**Conclusions:**

The magnitude of idiopathic scoliosis was relatively low in study setup. More attention is needed towards sitting furniture designs and for children reporting spinal pain.There is a reasonable level of recommendation to advocate that large school-based scoliosis screening was able to detect scoliosis among school children.

**Supplementary Information:**

The online version contains supplementary material available at 10.1186/s13690-021-00633-0.

## Background

Scoliosis refers to lateral curvature of the spine in the frontal plane and three-dimensional tensional deformity of both spine and trunk [1]. Vertebral rotation is a key distinguishing feature in the diagnosis of structural scoliosis [2, 3]. Physical examination for scoliosis mainly implies Adam’s forward bend test, the children stand and bend forward at the waist, with the examiner assessing for symmetry of the back from behind and beside the patient. Scoliosis will have a lateral bending of the spine but the curve will cause spinal rotation and a rib hump[4]. Early screening of scoliosis helps prevent progression to severe scoliosis and the need for surgical interventions [5]. The incredible advancement in the management of scoliosis and early detection had proven to be a very effective method in preventing scoliosis globally [6, 7].

The estimated global prevalence with the absence of data from some low and middle-income countries is 1 % [8, 9]. Though the global prevalence seems low, the complications of scoliosis are severe and life-threatening [10, 11]. School scoliosis screening can have sustained clinical effectiveness in identifying children with idiopathic scoliosis [12, 13]. The prevalence of idiopathic scoliosis is on the rise, scoliosis screening should be continued as a routine health service in schools or by general practitioners if there is no scoliosis screening policy [14].

Idiopathic scoliosis (IS) accounts for most cases of structural scoliosis when it is not due to diseases or injury to the musculoskeletal system [15]. In the USA, at least 3 % of the population has scoliosis and it is more likely that the rate is the same or even worse [16]. In idiopathic scoliosis(IS), Juvenile IS and adolescent IS develops at the age of 4–10 and 11–18 years, respectively [14, 17]. The systematic study in China reported that IS ranged from 0.26 to 2.5 % and the overall pooled prevalence was 1.02 % [18]. A Brazilian study of school children aged 10–14 years reported a prevalence of 1.4 % [19]. The prevalence of scoliosis among the general population worldwide ranged from 0.5 to 13 %, whereas among school populations, the prevalence has ranged from 0.5 to 3 % [20, 21].

Scoliosis screening is a broadly discussed topic especially among the school population, though arguments against screening do exist like; low predictive values leading to more referrals, possible increase to x-ray exposure in children, cost issue, and stress-induced by examinations [22]. Despite those facts, scoliosis screening is an important preventive measure to avoid deformity progression [23]. Evaluation of scoliosis by x-ray using Cobb angle or CT scan is the best evaluation, but implies a greater amount of radiation exposure and is not routinely performed.

Hence, the implication of accelerometer led scoliometer applications that mimics a scoliometer and are reasonably accurate, easy to use, cheap, acceptable, and more importantly lesser implications of radiationslike X-ray might optionally prove to be a better outcome screening tool [24–26]. While considering the concerns of radiation exposure for frequently monitoring IS among children and unaffordability of purchasing handheld standardized plastic scoliometer devices, scoliguage iPhone app device is an effective screening option particularly in the low and middle-income countries [27, 28]. Considering the resource constrain of physical clinical scoliometer devices in this country and increasing availability of smartphone, these angle measuring apps assume special importance of being cost-effective tools [3, 29]. The scoliguage app has a sensitivity of 85 % and specificity of 86 % at an angle of trunk rotation (ATR) cut off of 7 degree [30, 31]. Both studies proved scoligauge iPhone app to be valid against the scoliometer in clinical and controlled non-clinical settings. Smartphone aided ATR measurement had shown good reliability and accuracy with the clinical scoliometer [2, 32].

Lack of spinal screening and access to basic medical care in the low- and middle-income countries has led to severe spine deformities in children. Evidence reported that in the absence of effective screening programs, corrective exercise, and surgical management of scoliosis can lead to permanent deformity, pain, lung function compromise, low self-esteem, and neurological damage [33]. Only a few lucky children identified by NGO’s (American Jewish Joint Distribution Committee) are treated medically or surgically in capital Addis Ababa or abroad [34, 35].

There is a complete dearth of literature or data on idiopathic scoliosis on any population or age group in developing countries including Ethiopia. It is the appropriate time to begin several regional scoliosis screening programs initially to identify children with lateral spine deviations and develop further plans to prevent progression or treat spine deviations. This study aimed to identify the magnitude and associated factors of idiopathic scoliosis among school children by using non- invasive and less expensive scoliometer Smartphone app and Adams forward bending test (AFBT). Therefore, the main focus of this study was to screen screen idiopathic scoliosis based on ATR measures and provide preliminary data about idiopathic scoliosis among school children. .

## Materials and methods

### Study design and setting

A school-based cross-sectional survey was carried out as a part of the Gondar school children Scoliosis screening Program (GSSP) from March 2019 to June 2019. The study was done at seven elementary schools at Gondar. The study area has a latitude and longitude of 12º36 N 37º28 E with an elevation of 2133 m above sea level and average sunlight 12 h/day. According to the Ethiopian Demographic and Health Survey (EDHS, 2016), about 70 % of children attend school by age 7. Between age 8 and 13, more than 60 % of children attend school in Ethiopia [36, 37]. The EDHS 2016, reported a country-wide prevalence of stunned children under age 5 to be 38 %.

### Study participants and data collection procedures

Totally 1905 students from seven primary schools were screened for IS under the screening program by the Department of Physiotherapy. Among the included children, there were 961 boys and 944 girls, age range from 5 to 16 years. All primary schools in the town were enrolled, with no special consideration for geographical or economic representation. School children with the ability to ambulate independently, able to stand wearing the school bag, and able to bend forward to perform AFBT were included. Children ambulating using a wheelchair, mobility aids, and presenting with known congenital or structural deformities were excluded. Informed consent was obtained from parents/caregivers and school teachers, and assent was obtained from participating children. The screening program was carried out by physiotherapists working in musculoskeletal unit.

The data collection tool used for this study was the scoligauge app (Ockendon Partners Ltd, UK) created for iPhone (Apple Inc., Cupertino, CA) to mimic a scoliometer, with the price of only 0.99 US dollar, and measures the ATR. The iPhone 4 aided scoligauge app used for this study was tested for the intra-rater and inter observer reliability of ATR for the upper, lower thorax, and lumbar spine (ICC = 0.871 to 0.932) [38]. A validated correlation exists between the ATR angle and the radiograpghic Cobb angle. The screening examination began with the children standing straight with barefoot, with their back towards the physiotherapist, head up and the arms relaxed at the sides. With the children in this position, inspections such as shoulder asymmetries, scapula prominence, unequal waistline or arm distances, and lower limb length inequality were done and deviations identified were documented in the structured questionnaire (Additional file 1). Female children were using a specific top that allowed exposure of their back, their hair was tied up, and male children were topless. The children were instructed to perform Adam’s forward bend test while the testers placed the smart-phone on the thoracic or lumbar spine. The smart phone with the scoliometer HD App was placed on the apex of the lower (lumbar) middle (thoracic-lumbar) and upper (thoracic) back to measure the angle of trunk rotation. The same sets of mobile phones were used throughout phase one screening. Both the readings of smart phone scoliometer HD app and the findings of AFBT were recorded. The ATR finding is the primary outcome of interest and in addition the AFBT and spinal observation findings were presented as discrete variables. Their parents were also informed about the findings and also those with a scoliometer HD App reading > 7^o^ or positive AFBT were referred to the University of Gondar referral hospital for further screening and clinical examination. This study used the Bunnell screening cut-off criteria of scoligauge with ATR 0º to 3º - normal limits of trunk rotation, ATR 4º to 6º - intermediate trunk rotation, ATR ≥ 7º relevant or critical trunk rotation and high probability that the child has scoliosis. Idiopathic scoliosis was defined as ATR value (< 7^o^ and ≥ 7^o^, categories: spinal symmetry versus spinal asymmetry) in atleast one of the spinal regions (thoracic, thoracolumbar, & lumbar) and ATR measure is used as the outcome variable [39].

#### Statistics and data analysis

Data were coded and entered into Epi Info software version 7.0 and IBM Statistical Package for Social Sciences (SPSS) version 23 for Windows for statistical analyses [40]. Descriptive statistics were used to describe the sociodemographic characteristics, pain-related factors, and children-related factors. To assess the association between predictor variables and outcome variable (ATR ≥ 7º), binary logistics regression model was used. Predictor variables included in the regression models were age (categorized 5–10 and 11–16), school type (governmental and private), transport mode (walking, school bus transport, and public transport), duration of walking (categorized < 20 min and ≥ 20 min), way of carrying school supplies (backpack, shoulder strap and in hand), percentage of school bag’s weight of body weight (categorized 0–10 %, 10–20 %, > 20 %). Results were considered statistically significant when 95 % confidence intervals not containing unity (equal to *p-*value < 0.05) for both main effects and interaction effects. Initially, univariate analyses were conducted and predictor variables that were found statistically significant were entered into multivariate analysis. This study was reported in accordance with the STROBE reporting guidelines (Additional file 2).

## Results

### Study participant characteristics

In this study, a total of 1905 children constituted to the study subject who were aged between 5 and 16 years from seven elementary schools including four governmental schools (*n* = 1289; 67.7 %) and three private schools (*n* = 616; 32.3 %). The mean age of the participants was 11.6 years (SD 2.6 years), 33.4 % (*n* = 636) were under the age of 11 years old, and 49.6 % (*n* = 944) were female. More descriptive characteristics of the school children are presented in Table 1.

**Table 1:** Descriptive characteristics of the participant and distribution of idiopathic scoliosis among school children, Gondar; Ethiopia; *n* = 1905

Variables	Sample	Idiopathic scoliosis	
		Yes (asymmetry)	No (symmetry)
	N (%)	N (%)	N (%)
All participants	1905(100%)	62(3.3%)	1843(96.7)
Age (in years) m ean (11.6^+_^2.6)			
5-10	636(33.4%)	19 (30.6%)	617 (33.5%)
11-16	1269(66.6%)	43 (69.4%)	1226(66.5%)
Sex			
Male	961(50.4%)	35(56.5%)	926 (50.2%)
Female	944(49.6%)	27(43.5%)	917 (49.8%)
Height (in cm) as mean (SD)	132.4(12.02)	131.9(13.2)	135.1(14.4)
Weight (in kg) as mean (SD)	31.68(7.67)	29.7(7.5)	31.5(7.9)
Type of school			
Governmental	1289(67.7%)	44(70.9%)	1245(67.6%)
Private	616(32.3%)	18(29.1%)	598(32.4%)
School grade			
Grade 1-4	903(47.5%)	29(46.8%)	874(47.4%)
Grade 5-8	1002(52.5%)	33(53.2%)	969(52.6%)
Mode of Transport			
Walking	1067(56.0%)	45(72.6%)	1022(55.5%)
School bus	139(7.3%)	6(9.7%)	133(7.2%)
Public transport	699(36.7%)	11(17.7%)	688(37.3%)
Walking duration (*n*=1067)			
<20 minute	621(58.2%)	28(62.2%)	593(58.0%)
≥20minute	446(41.8%)	17(37.8%)	429(42.0%)
Carrying school supplies			
Backpack	838(44.0%)	25(40.3%)	813(44.1%)
Shoulder Single strap	708(37.2%)	24(38.7%)	684(37.1%)
In Hand	359(18.8%)	13(21.0%)	346(18.8%)
Preference of single strap (*n*=708)			
Right shoulder	519(27.2%)	15(24.1%)	504(27.3%)
Left shoulder	300(15.7%)	13(21.0%)	287(15.6%)
Both shoulders together	706(57.1%)	21(33.9%)	706(57.1%)
Bag weight in % of body weight			
0-10%	848(44.5%)	33(53.2%)	815(44.2%)
11-20%	832(44.7%)	21(33.9%)	811(44.0%)
> 20%	225(11.8%)	8(1.9%)	212(11.8%)
Furniture used in the class			
Chair with back rest	237(12.4%	3(4.8%)	234(12.7%)
Stool	177(9.3%)	8(12.9%)	169(9.2%)
Bench with back rest	447(23.5%)	16(25.8%)	431(23.4%)
Bench without back rest	1044(54.8%)	35(56.5%)	1009(54.7%)

### Pain profile and postural assessment of the school children

Five hundred and sixteen (*n* = 516, 27.1 %) students reported having experienced musculoskeletal pain in the spine during the idiopathic scoliosis screening period. Most of the children reported spinal pain in one segment (56.2 %, n 290) followed by two spinal segments (43.8 %, n 226). The most frequently recorded spinal segment of experienced musculoskeletal pain was the lumbar (34.98 %, *n* = 180) followed by the thoracolumbar region (27.5 %, *n* = 142) and the least testified musculoskeletal pain on the spinal segment was the cervical (6.9 %, *n* = 36). An overview of the spinal pain and postural description of the children is presented in Table 2.

**Table 2:** Pain and postural assessment characteristics of the school children, Gondar, Ethiopia (*n* = 1905)

Variables	Frequency(n)	Percent (%)
Severity of spinal pain (PNS)	*n* = 516	
Mild (1–3)	204	39.5
Moderate (4–6)	307	59.6
Severe (7–10)	5	0.9
Shoulder level equal		
Yes	1640	86.1
No	205	13.9
Arm to body space equal		
Yes	1850	97.1
No	55	2.9
Scapula level equal		
Yes	1878	98.6
No	27	1.4
Pelvic obliquity equal		
Yes	1892	99.3
No	13	0.7
Waist crease symmetry		
Yes	1894	99.4
No	11	0.6
Chest wall		
Normal	1856	97.4
Barrel	25	1.3
Pigeon	21	1.1
Funnel	3	0.2
Spine observation		
Normal	1707	89.6
Scoliosis right thoracic	3	0.2
Scoliosis left thoracic	8	0.4
Kyphosis thoracic	39	2.0
Lordosis lumbar	148	7.8
Leg length discrepancy		
Yes	5	0.3
No	1900	99.7
Adam’s forward bend test		
Gibbus +	34	1.8
Gibbus –	1871	98.2

*PNS *Pain Numbering Scale, + positive, - negative

#### Burden of Idiopathic scoliosis among school children

Among the 1905 school children screened, 62 (3.3 %) of them had ATR reading ≥ 7^0^ (positive finding) in atleast one spinal segment when screened using the scoliometer HD app with 95 % CI (2.5–4.1), among them 21(33.87 %) had very large scoliometer reading with ATR greater than 10 degrees, and of those sixty two children only 34 (1.8 %) were positive to Adam’s forward bend test. The categorized angle of trunk rotation in the different spinal segment with scoligauge application is mentioned in (Table 3).

**Table 3:** The angle of trunk rotation (ATR) in the different spinal segments with scoligauge application among school children, Gondar, Ethiopia (*n* = 1905)

ATR value	Frequency	Percent (%)
Upper spine (thoracic)		
< 7 degrees	1861	(97.7 %)
≥ 7 degrees	44	(2.3 %)
Middle spine(thoracolumbar)		
< 7 degrees	1882	(98.8 %)
≥ 7 degrees	23	(1.2 %)
Lower spine(lumbar)		
< 7 degrees	1889	(99.2 %)
≥ 7 degrees	16	(0.8 %)
ATR value ≥ 7 degrees (atleast one region)	62	(3.3 %)

#### Regression analysis and Factors associated with idiopathic scoliosis

In the univariable logistic regression analysis BMI, mode of transport, types of sitting furniture, school grade, and spinal pain were significantly associated with idiopathic scoliosis. But, in multivariable regression analysis students who have spinal pain and type of sitting furniture had significantly associated with idiopathic scoliosis *p* < 0.05 (Table 4).

The odds of developing idiopathic scoliosis in school children who had used stool for sitting 7.03 times (AOR: 7.03; 95 % CI: 1.52, 32.5) and who had used siting bench with back rest 4.12 times (AOR: 4.12; 95 % CI: 1.12, 15.14) is higher as compared to children who had used sitting in chair with back rest. The odds of having idiopathic scoliosis is 4.10 times (AOR: 4.10; 95 % CI: 2.42, 6.89) higher among those who had spinal pain complaint.

**Table 4:** Results of univariate (COR) and multivariate logistic regression of factors associated with idiopathic scoliosis among school children, Gondar, Ethiopia (*n* = 1905)

Variable	COR (95 % CI)	*P-value*		AOR (95 % CI)	*P-value*
Age					
5–10 years	1 ref	-		1	-
11–16 years	1.14 (0.66, 1.97)	0.64		1.61 (0.84,3.09)	0.15
**Gender**					
Male	1	-		1	-
Female	1.28 (0.77, 2.14)	0.34		1.30 (0.77, 2.21)	0.30
Bag/body weight					
0–10 %	1	-		1	-
11–20 %	1.09 (0.50, 2.41)	0.82		1.02 (0.57, )1.84	0.9
> 20 %	0.7 (0.31, 1.61)	0.40		1.11 (0.55, 2.23)	0.77
Types of chair used for sitting					
Chair with backrest	1	-		1	-
Stool	0.37 (0.11, 1.21)	0.10		7.03 (1.52, 32.5)	0.01^*^
Bench with backrest	1.37 (0.62, 1.95)	0.14		4.12 (1.12, 15.14)	0.03^*^
Bench without back rest	1.07 (0.58, 1.95)	0.13		4,56 (1.20, 17.34)	0.02^*^
School grade					
1–4	1.15 (0.68, 1.94)	0.61		1.42 (0.77, 2.65)	0.26
5–8	1	-		1	-
Mode of transport					
Walking	2.13 (1.21, 3.74)	0.01		1.44 (0.76, 2.70)	0.25
Motorized	1	-		1	-
Type of school					
Private	1	-		1	-
Governmental	1.17 (0.67, 2.05)	0.57		1.62 (0.76, 3.46)	0.21
Pain in the spine					
No	1	-		1	-
Yes	3.67 (2.19, 6.13)	0.001		4.10 (2.42, 6.89)	0.00^*^
Way of carrying school supplies					
Backpack	1	-	1		-
Shoulder strap	1.14 (0.64, 2.02)	0.65	1.02 (0.57, 1.84)		0.9
In hand	1.22 (0.62, 2.42)	0.56	1.11 (0.55, 2.23)		0.76

* variables significant with *p*-value ≤ 0.05, 1 = reference category, *COR *crude odds ratio, *AOR *adjusted odds ratio, *CI *confidence interval

## Discussion

The main intention of this study was to find out the burden of idiopathic scoliosis in school children and predictors associated with it. The burden of idiopathic scoliosis in school children was considerably low in this study. Complain of spinal pain, ways and material of sitting were independent predictors of scoliosis. The overall magnitude of idiopathic scoliosis in school children was 3.3 % with 95 % CI (2.5–4.1) when examined with the scoligauge application. This study found that about 1.8 % of the school children who were screened had positive findings for Adam’s forward bend test and 3.3 for scoligauge app with reading of ≥ 7º.

The prevalence reported in this study is however on the lower spectrum of the overall prevalence globally among children ranging from 0.35 to 13 % [41, 42]. Lack of previous regional data limits the comparison of the findings from this study in the local context. However, The prevalence of idiopathic scoliosis found in the current study is comparable to the finding of studies done in Australia (4.3 %) [43], and India (3.3 %) [16]. On the other hand, the finding of this study have higher or lower prevalence rates as compared with others studies. For example, across-sectional study done in South Africa reported that the prevalence of idiopathic scoliosis among school children was (8.2 %). A Brazilian study of school children aged 10–14 years reported a prevalence of 1.4 % [16].And in China, a systematic review reported that the prevalence of IS ranged from 0.26 to 2.5 % and the overall pooled prevalence was 1.02 % [18]. Potential explanations for different prevalence rates in these studies might be due to disparities in the nutritional status of children, socioeconomic status, the sample size, age variation, environmental factors and facilities and another main reason might be the difference in the outcome measurement tool like plastic scoliometer, scoliometer HD app, cobb’s angle, and Adam forward bending test. Besides, studies also report that the magnitude of IS drops at latitudes approaching the equator [10].

Surprisingly, the prevalence of IS in this study is almost similar for boys and girls, and the probable reasons might be the equal proportion both genders included in this study, variations in growth spurt, and puberty of the Ethiopian girls in comparisons to children elsewhere [26, 44].Nevertheless, similar to many studies, the trend of higher prevalence was found with age in this study [26]. Evidence suggests that the rate of development of spinal curvature changes rapidly at the beginning of puberty and there is a lack of consensus about the optimal age to start scoliosis screening. Hence, it is very essential to target children at risk, determine optimal age groups of Ethiopian school children based on their growth rate and puberty age for screening.

The predictors that were found to be significantly associated with spinal asymmetry from the logistic model were the poor design of school furniture and self-reported spinal pain. Studies report that children spend considerable time (80–85 %) sitting at school, which might exert an unhealthy load on the spinal structure over an extended duration and children at risk like; at puberty age, malnourished or bone mineral deficiency are vulnerable [45]. The association of spinal asymmetry with spinal pain in this study is suggestive that children with scoliosis adopt different postures in the sitting position which favor’s asymmetrical loading of the spine causing further damage to the spinal structures leading to pain [10].Gradually appearing mild scoliosis curvature usually do not cause pain, perhaps the reporting of spinal pain could have been due to poorly designed school furniture’s.

### The Strength and Limitation of the Study

We have used a validated measurement tool that has excellent inter-observer and intra-observer reliability. Seeing the benefits of upcoming research, there are undoubtedly few limitations to be mentioned. The lack of consent and approval for radiographic diagnosis did not allow an estimate of false-positive rates. Furthermore, the associated predictors using the regression model were based on few diagnosed cases and possible social bias of children while reporting pain. Hence, the findings should be deduced with cautiousness. For future researchers, after identifying children with spinal asymmetry by physical examination or scoligauge, it is better to use x-ray for confirmation and diagnosis of IS.

## Conclusions

The magnitude of idiopathic scoliosis and spinal asymmetry was relatively low in the study setup. Spinal pain, ways of sitting and sitting material were independent risk factors for idiopathic scoliosis in school children. School scoliosis screening programme was able to detect scoliosis and is also a crucial preface of preventive measures to avoidance of deformity progression.

## Supplementary information

**Additional file 1.**

**Additional file 2.**

## Data Availability

All data relevant to our findings are contained within the manuscript. Requests for further details on the dataset and queries concerning data sharing shall be arranged based on a reasonable request to mogesgashaw1@gmail.com.

## Abbreviations

AOR: Adjusted Odds Ratio
App: Application
CBR: Community Based Rehabilitation
CI: Confidence Interval
COR: Crude Odds Ratio
IS: Idiopathic Scoliosis
KM: Kilo Meter
NGO: Non-Governmental Organisation
SD: Standard Deviation
USA: United State of America
GSCSSP: Gondar School children Scoliosis Screening program

## References

[1] Çolak TK (2015). Scoliosis screening results of primary school students (11–15 years old group) in the west side of Istanbul. Journal of physical therapy science.

[2] Bonagamba GH, Coelho DM, Oliveira d (2010). Inter and intra-rater reliability of the scoliometer. Braz J Phys Ther.

[3] Izatt MT, Bateman GR, Adam CJ (2012). Evaluation of the iPhone with an acrylic sleeve versus the Scoliometer for rib hump measurement in scoliosis. Scoliosis.

[4] Reamy BV, Slakey J (2001). Adolescent idiopathic scoliosis: review and current concepts. Am Family Phys.

[5] Weinstein SL (2008). Adolescent idiopathic scoliosis. Lancet.

[6] Shen FH (2013). Pelvic fixation for adult scoliosis. Eur Spine J.

[7] Gaines R (2003). Tremendous advances continue in the surgical management of spinal deformity. Spine.

[8] Agel J, Akesson K, Johnell O, Amadio PC, Anderson M, Badley E, Balint G, Bellamy N, Bigos S, Bishop N, Bivans B. The burden of musculoskeletal conditions at the start of the new millennium. Technical Report Series. Geneva: World Health Organization; 2003 (919).

[9] Yip W, Hafez R, World Health Organization. Improving Health System Efficiency: Reforms for improving the efficiency of health systems: lessons from 10 country cases. World Health Organization; 2015 (No.WHO/HIS/HGF/SR/15.1).

[10] Grivas TB (2006). Association between adolescent idiopathic scoliosis prevalence and age at menarche in different geographic latitudes. Scoliosis.

[11] Schwab F (2006). A clinical impact classification of scoliosis in the adult. Spine.

[12] Negrini S (2012). 2011 SOSORT guidelines: orthopaedic and rehabilitation treatment of idiopathic scoliosis during growth. Scoliosis.

[13] Mirtz TA (2005). Adolescent idiopathic scoliosis screening for school, community, and clinical health promotion practice utilizing the PRECEDE-PROCEED model. Chiropractic Osteopathy.

[14] Fong DY (2015). A population-based cohort study of 394,401 children followed for 10 years exhibits sustained effectiveness of scoliosis screening. The Spine Journal.

[15] Wynne-Davies R (1968). Familial (idiopathic) scoliosis: a family survey. J Bone Joint Surg Br Vol.

[16] Kakar RS (2017). Review of physical activity benefits and potential considerations for individuals with surgical fusion of spine for scoliosis. International journal of exercise science.

[17] Zheng Y (2016). Prevalence and Determinants of Idiopathic Scoliosis in Primary School Children in Beitang District, Wuxi, China. J Rehabil Med.

[18] Rolton D, Nnadi C, Fairbank J (2014). Scoliosis: a review. Paediatrics Child Health.

[19] Salas MMS (2017). Prevalence and Associated Factors of Tooth Erosion in 8-12-Year-Old Brazilian Schoolchildren. Journal of Clinical Pediatric Dentistry.

[20] Karimian A (2015). Prevalence of scoliosis and associated risk factors in children and adolescents: a systematic review. Journal of Mazandaran University of Medical Sciences.

[21] Zhang H (2015). Prevalence of scoliosis among primary and middle school students in Mainland China: a systematic review and meta-analysis. Spine.

[22] Bakker N (2017). Promoting school efficiency: Dutch school doctors and the meaning of child health (1930–1970). Social Education History.

[23] Hresko MT, Talwalkar VR, Schwend RM. Position statement-screening for the early detection for idiopathic scoliosis in adolescents. SRS/POSNA/AAOS/AAP Position Statement. Scoliosis Research Society. 2015.

[24] Sarwark JF, Davis MM (2018). Evolving recommendations for scoliosis screening: a compelling need for further research. Jama.

[25] Franko OI, Bray C, Newton PO (2012). Validation of a scoliometer smartphone app to assess scoliosis. Journal of Pediatric Orthopaedics.

[26] Suh S-W (2011). Idiopathic scoliosis in Korean schoolchildren: a prospective screening study of over 1 million children. European spine journal.

[27] Howell JM, Craig PM, Dawe BG. Problems in scoliosis screening. Canadian Journal of Public Health/Revue Canadienne de Sante'e Publique. 1978;293-301. 688160

[28] Bunge EM (2006). Screening for scoliosis: do we have indications for effectiveness?. Journal of medical screening.

[29] Naziri Q, Detolla J, Hayes W, Burekhovich SA, Merola A, Akamnanu C, Paulino CB. A systematic review of all smart phone applications specificallyaimed for use as a scoliosis screening tool. J Long Term Eff Med Implants. 2018;28(1). 10.1615/JLongTermEffMedImplants.2017020737 29772989

[30] Franko OI, Tirrell TF (2012). Smartphone app use among medical providers in ACGME training programs. Journal of medical systems.

[31] Driscoll M (2014). Evaluation of an apparatus to be combined with a smartphone for the early detection of spinal deformities. Scoliosis.

[32] Qiao J (2014). Inter-and intraobserver reliability assessment of the axial trunk rotation: manual versus smartphone-aided measurement tools. BMC Musculoskelet Disord.

[33] Dye TD (2010). Complex care systems in developing countries: breast cancer patient navigation in Ethiopia. Cancer: Interdisciplinary International Journal of the American Cancer Society.

[34] Harris B (2015). Investigating the Universality of Preoperative Health Related Quality of Life (HRQOL) for Surgically-Treated Spinal Deformity in Young Adults: A Propensity Score Matched Comparison between African and United States Populations. The Spine Journal.

[35] Geck MJ, Singh D, Gunn H, Stokes JK, Truumees E. Is preoperative fibrinogen testing associated with total blood loss in adolescent idiopathic scoliosiscorrection?. Spine Deform. 2017;5(6):381-6. 10.1016/j.jspd.2017.05.001 29050713

[36] Ethiopia C, Macro O (2016). Demographic and health survey 2016.

[37] Ababa A (2016). Federal Democratic Republic of Ethiopia. TRANSPORT.

[38] Getnet MG, Jember G, Janakiraman B. Inter-and intra-observer reliability of scoliogauge app to assess the axial trunk rotation of scoliosis: Prospectivereliability analysis study. Int J Surg Open. 2020;27:5-9.

[39] Bunnell WP (2006). Reply: Selective Screening for Scoliosis. Clinical Orthopaedics Related Research®.

[40] George D, Mallery P. Descriptive statistics. In: IBM SPSS statistics 23 step by step. Routledge; 2016. pp. 126–34.

[41] Wong H-K (2005). Idiopathic scoliosis in Singapore schoolchildren: a prevalence study 15 years into the screening program. Spine.

[42] Adamczewska K (2019). The Angle of Trunk Rotation in School Children: A Study from an Idiopathic Scoliosis Screening. Prevalence and Optimal Age Screening Value. Int J Environ Res Public Health.

[43] Talasila SSA, Gorantla M, Thomas V (2017). A Study on Screening for Scoliosis among School Children in the Age Group of 10–14 Using a Cost Effective and an Innovative Technique. International Journal Of Community Medicine Public Health.

[44] Daruwalla J (1985). Idiopathic scoliosis. Prevalence and ethnic distribution in Singapore schoolchildren. J Bone Joint Surg Br Vol.

[45] Patias P (2010). A review of the trunk surface metrics used as Scoliosis and other deformities evaluation indices. Scoliosis.

