# Supplementary material for: Idiopathic scoliosis and associated factors among school children: a school-based screening in Ethiopia
Source: Arch Public Health. 2021 Jun 18;79:107. doi: 10.1186/s13690-021-00633-0 (PMC8212494; doi:10.1186/s13690-021-00633-0)
Supplement: Supplementary file 1 — Additional file 1. [file 13690_2021_633_MOESM1_ESM.docx]

**S1. Scoliosis screening tool-University of Gondar Hospital [English version] Unique ID:**

DATA COLLECTOR CODE: PIN: Students reg no:

| **Survey information** | | | | | | | | | | | | | | | | | | | | | |
| --- | --- | --- | --- | --- | --- | --- | --- | --- | --- | --- | --- | --- | --- | --- | --- | --- | --- | --- | --- | --- | --- |
|  |  | | | | | | **Response** | | | | | | | | | | | | | | **Code** |
| 1. | School name | | | | | |  | | | | | | | | | | | | | | S1 |
| 2. | Location of school [city] | | | | | |  | | | | | | | | | | | | | | S2 |
| 3. | Type of school | | | | | | Government0  Private1 | | | | | | | | | | | | | | S3 |
| 4. | Interviewer code/ID | | | | | |  | | | | | | | | | | | | | | S4 |
| 5. | Date and time of data collection | | | | | |  | | | | | | | | | | | | | | S5 |
| 6. | Grade of study | | | | | |  | | | | | | | | | | | | | | S6 |
| **Demographic data** | | | | | | | | | | | | | | | | | | | | | |
| 7. | Sex | | | | | | Female 0  Male 1 | | | | | | | | | | | | | | D1 |
| 8. | Age in years | | | | | |  | | | | | | | | | | | | | | D2 |
| 9. | Date of birth | | | | | |  | | | | | | | | | | | | | | D3 |
| 10. | Parents education level | | | | | | Father:  Mother: | | | | | | | | | | | | | | D4 |
| 11. | Parents occupation | | | | | | Father:  Mother: | | | | | | | | | | | | | | D5 |
| **Physical measurements** | | | | | | | | | | | | | | | | | | | | | |
| 12. | Standing height (cm) | | | | | |  | | | | | | | | | | | | | | P1 |
| 13. | Sitting height (cm) | | | | | |  | | | | | | | | | | | | | | P2 |
| 14. | Body weight with school bag (kg) | | | | | |  | | | | | | | | | | | | | | P3 |
| 15. | BW without school bag(kg) | | | | | |  | | | | | | | | | | | | | | P4 |
| **School supplies and Transport** | | | | | | | | | | | | | | | | | | | | | |
| 16. | Type of bag used to carry school supplies | | | | | | Backpack 1  Side/shoulder strap 2  In hand 3 | | | | | | | | | | | | | | T1 |
| 17. | If, backpack, carrying preference | | | | | | Right shoulder 1  Left shoulder 2  Both shoulder 3 | | | | | | | | | | | | | | T2 |
| 18. | Sitting furniture in school | | | | | | Chair with back rest 1  Stool 2  Bench with back rest 3  Bench without back rest 4 | | | | | | | | | | | | | | T3 |
| 19. | Time spent in playing/week in school | | | | | | <100 1  105-120 min 2  122-135 min 3  140-180 min 4  >190 min 5 | | | | | | | | | | | | | | T4 |
| 20. | Mode of transport to and from school | | | | | | Bus 1  Public transport 2  By walk 3 | | | | | | | | | | | | | | T5 |
| 21. | If, by walk, time of carrying bag | | | | | | Min: | | | | | | | | | | | | | | T6 |
| **Pain profile** | | | | | | | | | | | | | | | | | | | | | |
| 22. | Do you have pain in your spine | | Yes 1No 2 | | | | | | | | | | | | | | | | | | A1 |
| 23. | If yes, location | | C | | T | | | | L | C&T | | | | C&L | | | C&T&L | | | T&L | A2 |
| 24. | NRS rating scale | |  | | | | | | | | | | | | | | | | | | A3 |
| **Postural assessment-Observation** | | | | | | | | | | | | | | | | | | | | |  |
| 25. | Shoulder level equal | Yes | | | | | | No | | | | | If no, **R L** | | | | | | | | O1 |
| 26. | Arm to body space equal | Yes | | | | | | No | | | | | If no, **R L** | | | | | | | | O2 |
| 27. | Scapula level equal | Yes | | | | | | No | | | | | If no, **R L** | | | | | | | | O4 |
| 28. | Pelvic obliquity equal | Yes | | | | | | No | | | | | If no, **R L** | | | | | | | | O5 |
| 29. | Waist crease greater | Yes | | | | | | No | | | | | If yes, **R L** | | | | | | | | O6 |
| 30. | Chest wall | 1. Normal | | | | | | 2.Barrel | | | | | 3.Pigeon | | | | | | 4.Funnel | | O7 |
| 31. | Spine observation | **0** | | Normal | | | | | | | | | | | | | | | | | O8 |
|  |  | **1** | | Scoliosis Tif yes side of convexity**R L** | | | | | | | | | | | | | | | | |  |
|  |  | **2** | | Scoliosis Lif yes side of convexity**R L** | | | | | | | | | | | | | | | | |  |
|  |  | **3** | | Kyphosis T | | | | | | | | | | | | | | | | |  |
|  |  | **4** | | Lordosis L | | | | | | | | | | | | | | | | |  |
| 32. | Chest expansion (cm) | XP level | | | | | | | | | | | | | Diap level | | | | | | O9 |
| 33. | Leg length inequality | Yes 1 No 2 | | | | | | | | | | | | | | | | | | | O10 |
| 34. | Leg length measurement | Right: cm | | | | | | | | | | Left: cm | | | | | | | | | O11 |
| 35. | Scober’s test |  | | | | | | | | | |  | | | | | | | | | O12 |
| **Primary outcomes** | | | | | | | | | | | | | | | | | | | | | |
| 35. | Adam’s FBT | | 1. Gibbus +ve | | | | | | | | 2. Gibbus–ve | | | | | | | | | | SC1 |
| 36. | Location of prominence | | 1. Thoracic | | | | | | | | 2. Lumbar | | | | | | | | | | SC2 |
| 37. | If yes, side of gibbus | | **R L** | | | | | | | | **R L** | | | | | | | | | | SC3 |
| 38. | Scoliometer reading [ATR]  Please specify site and side of prominence: | | **DC 1** | | | | | | | | | | | | | **DC2** | | | | | SC4 |
|  |  |  | **R L** | | | | | | | | | | | | | **R L** | | | | |  |
|  |  |  | **L** | | | 1. Reading one | | | | | | | | | | 1. Reading one | | | | |  |
|  |  |  | **L** | | | 2. Reading two | | | | | | | | | | 2. Reading two | | | | |  |
|  |  |  | **LT** | | | 1. Reading one | | | | | | | | | | 1. Reading one | | | | |  |
|  |  |  | **LT** | | | 2. Reading two | | | | | | | | | | 2. Reading two | | | | |  |
|  |  |  | **UT** | | | 1. Reading one | | | | | | | | | | 1. Reading one | | | | |  |
|  |  |  | **UT** | | | 2. Reading two | | | | | | | | | | 2. Reading two | | | | |  |
| 39. | Hand to ground distance | | Fingers touch ground: Yes | | | | | | | | | | | | | | | No | | | SC5 |
