# Supplementary material for: Idiopathic scoliosis and associated factors among school children: a school-based screening in Ethiopia
Source: Arch Public Health. 2021 Jun 18;79:107. doi: 10.1186/s13690-021-00633-0 (PMC8212494; doi:10.1186/s13690-021-00633-0)
Supplement: Supplementary file 2 — Additional file 2. [file 13690_2021_633_MOESM2_ESM.docx]

STROBE checklist for observational studies

**Title: Idiopathic scoliosis and associated factors: a study from school-based screening in Ethiopia: a cross-sectional study**

**STROBE** Statement—checklist of items that should be included in reports of ***cross-sectional studies***

|  | Item No | Recommendation |
| --- | --- | --- |
| **Title and abstract** | 1 | 1. Study design is indicated in the abstract, methods section as school based cross-sectional study design. |
|  |  | (b) A balanced summary of what was done and what was found is provided in the methods and result section of the abstract |
| Introduction | | |
| Background/rationale | 2 | The scientific background and rationale for the investigation is reported. The rationale of the study was reported on 5^th^ paragraph of background section |
| Objectives | 3 | The general objectives of this study were stated as the final sentence of last paragraph under background session & the second sentence of the abstract. |
| Methods | | |
| Study design | 4 | It is presented in the first sentence of first paragraph of method section |
| Setting | 5 | A detailed description of the study area, location, and participant, described in the first and second paragraph of method section. |
| Participants | 6 | The study participants are clearly stated in the 2^nd^ and 3^rd^ paragraph of methods session. |
| Variables | 7 | Both outcome and predictor variables are mentioned study participant and statics sub-section in the methods in the last paragraph. |
| Data sources/ measurement | 8 | Source of data and data analysis methods are discussed in the 5^th^ and 6^th^ paragraphs of methods session |
| Bias | 9 | Efforts to address potential sources of bias were described in several part of method session. |
| Study size | 10 | Not relevant since it a survey study |
| Quantitative variables | 11 | All quantitative variables treated as qualitative after categorizing them in one of most commonly used categories. |
| Statistical methods | 12 | (*a*) Statistical methods used in this study are described under data statistics sub-section in the last part of method session. |
|  |  | (*b*) Both sub group analysis and interaction terms were used. |
|  |  | (*c*) There were no missing data in this study |
|  |  | *(d)* Not applicable |
|  |  | (*e*) Not applicable |
| Results | | |
| Participants | 13 | (a) Number of participants is presented in the first paragraph of results session and detail socio-demographic characteristics in the table 1. |
|  |  | (b) . Not applicable |
|  |  | (c) This was cross-sectional study so; there is no flow as that of longitudinal study. |
| Descriptive data | 14 | (a) Characteristics of study participants (e.g. demographic, pain related variables,) and information on exposures and potential confounders is presented in tables 1 & 2. |
|  |  | (b) There were no missing data in this study. |
| Outcome data | 15 | Outcome variable (PPGP) described and summarized in table1. |
| Main results | 16 | (*a*) Unadjusted estimates and confounder-adjusted estimates and their precision (eg, 95% confidence interval) are presented in table 4. Discussed under regression analysis 1^st^ para in result section. |
|  |  | 1. Category boundaries of continuous variables were categorized and reported in all tables. |
|  |  | (*c*) Regression model was used and expressed in odds ratio in table 4. |
| Other analyses | 17 | No clear or significant sub group difference noted and interaction terms were used but non-significant. |
| Discussion | | |
| Key results | 18 | Key results to study objectives are discussed under discussion session with references. |
| Limitations | 19 | Limitations and possible strengths related to the current study are discussed in the strength and limitation session on the way of viewing direction for researchers. |
| Interpretation | 20 | A cautious overall interpretation of results considering objectives, results from similar studies, and other relevant evidence is discussed under limitation of discussion session. |
| Generalizability | 21 | Generalisability (external validity) of the study results are mentioned under conclusion section |
| Other information | | |
| Funding | 22 | Information regarding the source of funding (University of Gondar) and the role of the funders for the present study is presented under acknowledgment section. |
